# Green tea proanthocyanidins cause impairment of hormone-regulated larval development and reproductive fitness via repression of juvenile hormone acid methyltransferase, insulin-like peptide and cytochrome P450 genes in *Anopheles gambiae sensu stricto*

**DOI:** 10.1371/journal.pone.0173564

**Published:** 2017-03-16

**Authors:** Jackson M. Muema, Steven G. Nyanjom, James M. Mutunga, Sospeter N. Njeru, Joel L. Bargul

**Affiliations:** 1 Department of Biochemistry, Jomo Kenyatta University of Agriculture and Technology, Nairobi, Kenya; 2 Malaria Research Programme, International Centre of Insect Physiology and Ecology, Nairobi, Kenya; 3 Department of Medicine, Faculty of Health Sciences, Kisii University, Kisii, Kenya; 4 Molecular Biology and Bioinformatics Unit, International Centre of Insect Physiology and Ecology, Nairobi, Kenya; USDA Agricultural Research Service, UNITED STATES

## Abstract

Successful optimization of plant-derived compounds into control of nuisance insects would benefit from scientifically validated targets. However, the close association between the genotypic responses and physiological toxicity effects mediated by these compounds remains underexplored. In this study, we evaluated the sublethal dose effects of proanthocyanidins (PAs) sourced from green tea (*Camellia sinensis*) on life history traits of *Anopheles gambiae* (*sensu stricto*) mosquitoes with an aim to unravel the probable molecular targets. Based on the induced phenotypic effects, genes selected for study targeted juvenile hormone (JH) biosynthesis, signal transduction, oxidative stress response and xenobiotic detoxification in addition to vitellogenesis in females. Our findings suggest that chronic exposure of larval stages (L3/L4) to sublethal dose of 5 ppm dramatically extended larval developmental period for up to 12 days, slowed down pupation rates, induced abnormal larval-pupal intermediates and caused 100% inhibition of adult emergence. Further, females exhibited significant interference of fecundity and egg hatchability relative to controls (*p* < 0.001). Using reverse transcription quantitative polymerase chain reaction (RT-qPCR), our findings show that PA-treated larvae exhibited significant repression of *AgamJHAMT* (*p* < 0.001), *AgamILP1* (*p* < 0.001) and *AgamCYP6M2* (*p* < 0.001) with up-regulation of *Hsp70* (*p* < 0.001). Females exposed as larvae demonstrated down-regulation of *AgamVg* (*p* = 0.03), *AgamILP1* (*p* = 0.009), *AgamCYP6M2* (*p* = 0.05) and *AgamJHAMT* (*p* = 0.02). Our findings support that *C*. *sinensis* proanthocyanidins affect important vectorial capacity components such as mosquito survival rates and reproductive fitness thus could be potentially used for controlling populations of malaria vectors.

## Introduction

The mosquito *Anopheles gambiae* (*sensu stricto*) is the most dynamically evolving and efficient malaria vector in sub-Saharan Africa [1–3]. The ecological factors that prevail across countries in Africa and anthropogenic activities coupled with climate change affect vector dynamics, favoring malaria transmission to human hosts by these vectors, and vector control is nearly at cross roads [4–6]. Reduction of vectorial capacity below the critical threshold required to achieve a basic reproduction rate of less than 1 is the ultimate goal of vector control interventions [7]. Efforts to control these devastating disease-transmitting vectors using chemical insecticides have reduced global malaria incidences by 37% and 42% in Africa [8]. However, residual transmission is currently accounting for over 430,000 deaths reported worldwide in the year 2015 and approximately 3.3 billion people are at risk of contracting malaria due to increasing cases of insecticide resistance in mosquitoes, coupled with limitations of the existing control methods against malaria vectors [8, 9]. In light of these, interventions targeting the immature stages of mosquitoes that are susceptible to chemical attacks and with limited chances of developing resistance seem quite promising vector control approaches that could reduce malaria transmission rates in high transmission endemic regions [10, 11]. Indeed, several studies have shown direct consequential impacts of manipulative effect of larval ecology on life history attributes and capacity of emergent mosquitoes to transmit *Plasmodium falciparum* parasites [12–15].

Transmission of malaria to human hosts by female anopheline mosquitoes is influenced by their vectorial capacities and competence, evolutionary traits which are fine-tuned through interactions of genetic and environmental determinants acting directly on larval stages [16, 17]. In view of these, mosquito population dynamics^_^ the potential driver of epidemiological malaria transmission^_^ depends on various attributes such as fecundity [16], larval productivity [13], female mosquito longevity [18], climatic conditions, biotic and abiotic environmental fluctuations [17]. At the genetic level, the transcriptional regulation of various physiological processes that influence mosquito life history attributes and vector bionomics based on the sensory inputs from prevailing environmental conditions dictates the success of resultant vectors [19, 20]. Essentially, just like other insects, juvenile hormone (JH), insulin/insulin-like signaling (IIS), and cytochrome P_450_ monooxygenases interact to control various physiological processes that influence mosquito fitness and progeny [21]. These processes include metamorphosis, reproduction, behavioral response, stress tolerance, ageing, metabolism and morphogenesis [22–26]. During the juvenile stages, IIS signaling cascade sends nutritional signals to neurosecretory cells which then release developmental hormones for regulating the timing of metamorphosis [21, 22, 27, 28]. To regulate insect development between instars, JH binds to methoprene-tolerant (Met) protein transducing the ecdysone-induced signals into stage-specific responses [26]. The JH titers in hemolymph correlate with its biosynthetic activity in the *corpora allata* (CA) *via* regulatory signals of insulin-like peptides (ILPs). Accordingly, juvenile hormone acid methyltransferase (JHAMT), the rate-limiting catalytic enzyme in the JH biosynthetic pathway, is a key regulator of insect metamorphosis and its expression determines the JH titers in hemolymph [29, 30]. Therefore, interventions that interfere with JHAMT expression either through chemical exposure, RNA*i* silencing or genetic ablation of CA considerably perturbs precocious development, reproduction and/or induces morphogenetic aberrations [31]. For instance, inhibition of JHAMT transcription in *Aedes* caused larval mortality, delayed adult eclosion by 3 weeks and reduced egg production by 50% [32]. Soon after adult emergence and bloodmeal uptake, the IIS, JHAMT and Halloween-like cytochrome monooxygenases play important roles in vitellogenesis and oocyte maturation [22, 33, 34]. Inhibition of JHAMT and IIS pathway has been reported to negatively impact transcription of vitellogenin (Vg) genes and egg maturation in mosquitoes and *Tribolium castaneum* [35, 36]. Further, Sheng *et al*., [37] reported that RNA*i* silencing of JH biosynthesis and signaling elements resulted in reduced ILP and forkhead transcription factor O (dFOXO)-mediated transcription of vitellogenin in *T*. *castaneum*. Thus, interference of these regulatory feedback inputs under unfavorable conditions generates phenotypes exhibiting delayed molting, prolonged development, heterochronic larval-pupal intermediates, delayed maturity, reproductive diapause and sterility in many insects by conferring life history trade-offs [38]. In order to survive such conditions, mosquitoes express detoxification enzymes (cytochrome P_450_) and heat shock proteins, similar to other organisms subjected to xenobiotic stress [39, 40]. Therefore, we narrowed down these candidate genes (*AgamJHAMT*, *AgamILP1*, *AgamCYP6M2*, *Hsp70* and *AgamVg*) based on the induced phenotypes of mosquito larvae and adult females.

In pursuit of mosquito control that reduces the reliance on synthetic insecticides, researchers have revitalized interests in search of novel chemistries to control mosquitoes that have less harmful impacts on environmental health [41, 42]. As a result, evaluations of diverse plant compounds have shown direct toxicities and growth inhibitory properties [43–45] while others confer repellent properties [46]. Despite the rigorous research on bioactive phytocompounds, information regarding their molecular mechanisms of action, which could lead to production of selective, target-specific and resistance-resilient insecticides against malaria-transmitting mosquitoes, is still lacking for most of these compounds. However, attempts to elucidate their mechanisms of action in herbivorous insects have shown that these compounds preferentially interfere with digestive enzymes, signal transduction pathways, hormonal balance, membrane potential, nutrient and ion transport, hence limiting the chances for development of resistance [47].

Proanthocyanidins (PAs) are oligomeric or polymeric polyphenolic compounds popularly known as condensed tannins (CTs) formed through epimerization of catechins and epicatechins [48]. In plants, PAs constitute the second most abundant natural phenolic compounds after lignin [49]. PAs exhibit a broad spectra of biological and toxicological activities by targeting various processes [50]. In health care, PAs have been implicated in alleviation of oxidative stress related complications in cancer, cardiovascular diseases, diabetes, cataracts, ageing, obesity and neurodegenerative diseases due to their ability to scavenge free radicals, chelate metal ions, inhibit pro-oxidative enzymes and lipid peroxidation [51]. Further, PAs exhibit antimicrobial effects that inhibit adhesion of bacteria and viruses to host cells thus blocking subsequent proliferation [51–53]. Besides these health benefits in human beings, PAs play defensive roles in plants by producing pleotropic effects in insect herbivores that are detrimental to their growth and survival [49]. Emerging evidence indicates that PAs from *C*. *sinensis* leaf extracts have potential for suppressing mosquito vector populations through interference of larval survival and development [54].

With the aforementioned bioactivity of *C*. *sinensis* extracts against mosquito vectors, there is a paucity of information explaining the molecular basis for the observed physiological toxicity effects. The present study aimed to: 1) investigate the sublethal dose effects of PAs isolated from *C*. *sinensis* leaves (clone TRFK 6/8) on life history traits of *An*. *gambiae* (*s*.*s*.), and 2) correlate the resultant PA-induced phenotypic responses to the modulated gene expression profiles. Therefore, we exposed L3/L4 instars of *An*. *gambiae* (*s*.*s*.) to sublethal doses of PA-rich fractions and evaluated the effects on larval development and reproductive fitness in female adults. To analyze the molecular mechanisms of action conferred by these compounds, changes in gene expression profiles were assessed using reverse transcription quantitative polymerase chain reaction (RT-qPCR). Our findings show that the PA-rich fraction of *C*. *sinensis* leaf extracts dramatically protracted the larval developmental period, disrupted adult emergence, and remarkably reduced female fecundity and egg hatchability. These phenotypic characteristics were manifested through the modulation of gene expression associated with mosquito developmental and reproduction physiology.

## Materials and methods

### Ethical statement

All experimental procedures were conducted with compounds isolated from non-endangered plant species. No specific permissions were required for sample acquisition. However, authorization to use the plant for study was granted by Limuru Archdiocesan farmers. The mosquito blood feeding protocol (Ref: KEMRI/RES/7/3/1) was approved by the Kenya Medical Research Institute (KEMRI) and reviewed by the Institutional Animal Care and Use Committee (IACUC) at the International Centre of Insect Physiology and Ecology (*icipe*). In this study, five mice (*n* = 5) were used for mosquito blood feeding for five times. The mice received humane care in strict accordance with the Guide for the Care and Use of Laboratory Animals of National Institutes of Health (NIH). To minimize suffering, mice were anesthetized with intraperitoneal injection of 0.1 ml of 5% sodium pentobarbital then restrained using a wire mesh prior to exposure to mosquitoes for bloodmeal acquisition. The animals were returned to their cages after mosquito feeding.

### Maintenance of laboratory colony of mosquito

Mbita strain of mosquitoes used in this study was obtained from *icipe*’s Arthropod Rearing and Quarantine Unit (ARQU), Nairobi, Kenya. This strain was initially established at *icipe*’s Thomas Odhiambo campus near Lake Victoria in 2003. The larvae were reared under laboratory conditions of water temperature (28 ± 2°C), relative humidity of 65–80% and 12:12 h (light: dark) photoperiod. The larvae were reared in large plastic pans (37 × 31 × 6 cm) containing dechlorinated water at densities of 200–300 larvae per pan and fed on an artificial diet: Tetramin^®^ fish meal (Tetra GmbH, Melle, Germany). The larval breeding water was replaced with fresh water and diet every two days. Pupae were collected in plastic cups and transferred to standard rearing cages measuring 30 × 30 × 30 cm. Adults were provided with a 10% sucrose solution in a glass tube (2 × 8 cm) connected to a paper tube as a wick. Female mosquitoes were blood-fed on restrained Swiss albino mice after 3–4 days post-emergence and provided with oviposition plastic containers (11.5 cm in diameter and ~ 6.2 cm in depth, lined interiorly with a piece of filter paper as oviposition site) for egg collection 2–3 days after the bloodmeal. The eggs were seeded in dechlorinated water under insectarium conditions for colony cycle maintenance.

### Test insecticide

The proanthocyanidin-rich fraction was isolated from a 90% methanolic extract of *C*. *sinensis* leaves (clone TRFK 6/8) as previously described [54]. Briefly, 500 g of pulverized leaf powder was soaked in 2 L 90% methanol (Sigma Aldrich, St. Louis, USA) for 72 h with intermittent shaking after which the extract was filtered through a filter paper (Whatman Inc., Haverhill, USA) and excess solvent removed using a rotary evaporator (Laborota 4000 efficient, Heidolph, Germany). The residual extract was lyophilized in a freeze dryer (Labconco stoppering tray dryer, Labconco Corporation, USA). About 35 g of the dry biomass was fractionated into 11 fractions on a silica-packed column. The bioactive fraction was spectroscopically analyzed using UPLC/ESI-Qtof/MS and revealed an abundance of proanthocyanidins. Different concentrations of the PA-rich fraction were prepared by dissolving the respective amounts in 0.2% (*v/v*) ethanol (Fisher Scientific, Loughborough, UK) and then dispensed into 5 beakers per treatment dose.

### Experimental bioassays

#### (i) Acute toxicity and survival assays

In order to establish the optimal sublethal doses for subsequent experimental studies, toxicity assay of the PA-rich fraction was carried out following WHO guidelines for testing mosquito larvicides [55]. Batches of 20 L3/L4 instars in five replicates (*n* = 100) were separately put into 250-ml glass beakers containing 100 ml of the PA-rich fraction at specific concentrations of 12.5 ppm, 10 ppm, 5 ppm, 2.5 ppm and 1 ppm. The negative control was comprised of larvae treated with 0.2% (*v/v*) ethanol diluted in distilled water. The above assays were repeated three times under controlled laboratory conditions of water temperature 28 ± 2°C, relative humidity 65–80% and photoperiod of 12L:12D (L = light, D = dark). Larvae were fed Tetramin^®^ fish meal (Tetra GmbH, Melle, Germany) and mortality rates recorded from each treatment group every 24 h for 48 h.

Further, the survival rates of larvae chronically exposed to sublethal doses were analyzed as follows; batches of 20 L3/L4 instars in five replicates (*n* = 100) were separately put in beakers containing PA-rich fractions at concentrations of 1 ppm, 2.5 ppm and 5 ppm (below the LC_50_). The negative control group of larvae was treated under similar conditions as test samples, except with 0.2% (*v/v*) ethanol diluted in distilled water. The larvae were fed on 0.3 mg of Tetramin^®^ fish meal for the entire experimental period. The assay was carried out under controlled insectary conditions (water temperature 28 ± 2°C, relative humidity 65–80% and photoperiod of 12L:12D). Dead larvae were removed from the beakers every day and the number of surviving larvae was similarly recorded in each assay until the death of the last larva. These assays were replicated three times.

#### (ii) Effect of proanthocyanidin-rich fractions on development of mosquito larvae

Baseline data collection on mosquito development was performed by setting up three replicates of *An*. *gambiae* (*s*.*s*.) larvae (*n* = 10 larvae per replicate) in 250-ml beakers containing 100 ml distilled water under controlled rearing conditions of water temperature 28 ± 2°C, relative humidity 65–80% and photoperiod of 12L:12D (L = light, D = dark). Larvae (L1) were fed Tetramin^®^ fish meal. The time taken for development from one instar to the next was recorded as a mean value of the three replicates until pupal stages eclosed to adults.

**Fig 1:**
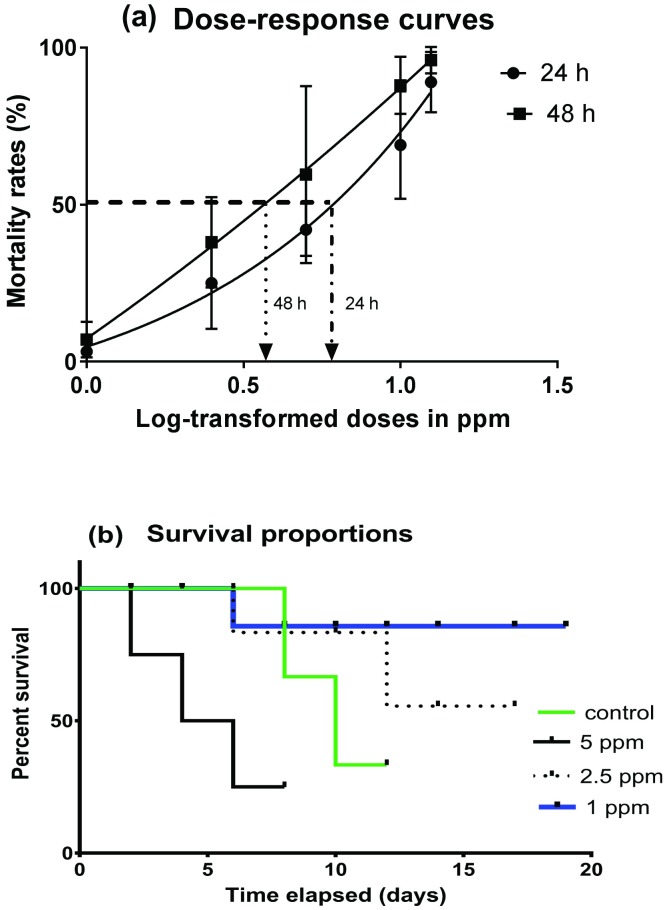
Effects of proanthocyanidin-rich fractions on survival of *An*. *gambiae* (*s*.*s*) larvae. **(a)**: Dose-response curves showing acute toxicity effect of PA-rich fractions on L3/L4 instars. L3/L4 instars were exposed to various doses of PA-rich fractions and mortality rates recorded every 24 h until 48 h post-exposure. Half-maximal lethal concentration (LC_50_) was established at 5.52 ppm for 24 h and 4.45 ppm for 48 h. **(b)**: Survival rates of L3/L4 instars on exposure to sublethal doses of PA-rich fractions relative to controls. Exposure of L3/L4 instars to sublethal doses of PA-rich fractions revealed dose-dependent survival rates with high survival rates exhibited in 1 ppm followed by 2.5 ppm, negative controls and least 5 ppm. Experimental data were collected from 3 independent replicates.

Optimal sublethal doses of the larvicide were based on dose-response curves generated from probit analysis (Fig 1a). Batches of 20 L3/L4 instars of *An*. *gambiae* (*s*.*s*.) in five replicates (*n* = 100) were exposed to PA-rich fractions at 1, 2.5 and 5 ppm following WHO guidelines for testing mosquito larvicides [55]. The control group was treated with a solution of 0.2% (*v/v*) ethanol (Fisher Scientific, Loughborough, UK) diluted in distilled water. The developmental period taken during the larval phase, rates of pupation and adult emergence, induced morphological defects and abnormal behavior were recorded. PA-induced developmental phenotypes were examined microscopically at a magnification of 25× using a Leica microscope equipped with Leica Application Suite version 3.0.0 for imaging (Leica Microsystems, Heerbrugg, Switzerland).

#### (iii) Effect of proanthocyanidin-rich fraction on reproductive viability of emergent female mosquitoes

The effects of chronic larval exposure on the reproductive fertility of adult mosquitoes that emerged from PA-treated water were assessed. Equal numbers of emerged males (*n* = 50) and females (*n* = 50) from PA-treated water and experimental controls were fed 10% sucrose solution, and allowed to mate with the same cohorts for 72 h. The female mosquitoes were provided a bloodmeal from five (*n* = 5) restrained Swiss albino mice 72 h post-emergence. Equal numbers of gravid females from the respective treated (*n* = 10) and control groups (*n* = 10) were replicated five times and then provided with oviposition plastic containers lined with wet filter papers for three consecutive nights. The number of eggs laid in each group was counted under a light microscope. Each set of eggs was separately seeded into 250-ml plastic containers containing fresh dechlorinated water, and egg hatching rates monitored every 12 h for 72 h. All the counts were compared between the test and control groups and expressed in percentages. The percentage reduction in oviposition, percentage hatchability, and percentage reduction in hatchability were calculated as follows:-

Percentage (%) reduction in oviposition=[1−(number of eggs laid in PA-treated group÷average number of eggs laid in control group)]×100

Percentage (%) hatchability=(number of larvae hatched÷total number of eggs introduced)×100

Percentage (%) reduction in hatchability=[1–(number of larvae hatched in PA-treated group÷number of larvae hatched in control group)]×100

### Total RNA isolation

Total RNA was isolated from 72 h-exposed larvae and from female adults 72 h after bloodmeal using TRIzol reagent according to the manufacturer’s instructions (Invitrogen, Carlsbad, CA). Thirty larvae and ten female mosquitoes from treated groups and their respective controls were separately homogenized in 750 μl of TRIzol reagent, vortexed and centrifuged for 2 min at 12000 ×g at 4°C for absolute solubilization of nucleoprotein complexes. For phase separation, 200 μl of molecular grade chloroform was separately added to each sample. Total RNA was precipitated following addition of 500 μl ice-cold isopropanol and the pellet washed twice with 200 μl of 75% ethanol (molecular grade). The RNA pellet was then re-solubilized in 50 μl RNase-free water and stored at -80°C until use. RNA purity was calculated based on A_260/280_ ratio, whereas the yield was assessed using Nanodrop spectrophotometer (Thermo Scientific, USA).

### First strand cDNA synthesis

Total RNA was treated with DNase I in order to remove any contaminating genomic DNA from the RNA sample. Synthesis of the first strand cDNA was performed using High Capacity cDNA Reverse Transcription kit (Applied Biosystems, USA) following manufacturer’s instructions. In a reaction volume of 10 μl, the reaction mixture was prepared using 1 ng of RNA template, 0.5 μl MultiScribe^®^ MuLV reverse transcriptase, 0.5 μl RNase inhibitor, 1× dNTPs mix, 1× reaction buffer, 1× RT random primers and brought to final reaction volume with 1.6 μl nuclease-free water. PCR-based reverse transcription reaction was set at 25°C for 10 min, 37°C for 120 min and 85°C for 5 min as recommended by manufacturer. The cDNA was stored at -30°C ready for use in RT-qPCR analysis.

### Quantitative RT-PCR analysis

Gene-specific primers targeting probable responsive genes, based on the induced mosquito phenotypes following exposure to PA-rich fraction, were designed *in silico* using Primer3 version 4.0.0. The primer sequences were synthesized by GenScript (GenScript Biotech Corporation, USA). Gene-specific primers are listed in Table 1 below.

**Table 1:** RT-qPCR gene-specific primer sets. The primers target genes for signal transduction, stress response, xenobiotic metabolism, JH biosynthesis, vitellogenesis and endogenous reference genes ribosomal s7 protein, α-tubulin.

Gene	Accession number	Primer sequences (5'-3')	Product size (bp)
**Insulin-like peptide gene 1**	**AGAP010605**	Fwd **GCTTCTGCTCGTTCTGCTCT**Rev **ACGCCAACGGTATTCTGAAC**	152
**Heat shock protein 70kDa**	**AGAP004581**	Fwd **ACGCCAACGGTATTCTGAAC**Rev **ACAGTACGCCTCGAGCTGAT**	197
***CYP6M2***	**AGAP008212**	Fwd **AGGTGAGGAGAGTCGACGAA**Rev **ATGACACAAACCGACAAGG**	235
**JH acid methyltransferase**	**AGAP005256**	Fwd **GAAGGGCTGGACAATTTGAA**Rev **TTCTCTTCGCTGCCAGTTTT**	146
**Vitellogenin**	**AGAP004203**	Fwd **CAGCCCTTCGTCTACTACGC**Rev **AAC TCGAGGGCACTGGA**	245
**Ribosomal S7 protein(internal reference)**	**AGAP010592**	Fwd **GCTGTTAGAAGCGGGGGAAT**Rev **CGGCCAGTCAGCTTCTTGTA**	183
**α-tubulin (internal reference)**	**AGAP010971**	Fwd **CAATGAGGCGATCTACGACA**Rev **TACGGCACCAGATTGGTCT**	171

Fwd: Forward primer, Rev: Reverse primer

Gene expression studies were performed on a Stratagene Mx3005P real time PCR machine (Agilent technologies, USA) using 1.042× SYBR^®^ Green master mix (Thermo Scientific, USA), 10-fold diluted cDNA template and 0.5 picomoles for forward and reverse primers in a total reaction volume of 12.5 μl. RT-qPCR amplification profile comprised of: a denaturation step of 95°C for 10 min, 40 cycles of (95°C for 30 sec, 60°C for 30 sec and 72°C for 30 sec) and a final dissociation cycle of 95°C for 1 min, 55°C for 30 sec and 95°C for 30 sec. All amplification reactions were conducted in triplicates and the mean C_T_ values computed for each gene. Normalization of gene expression levels was carried out using ribosomal s7 and α-tubulin as the internal references. The fold changes in expression levels were calculated using 2^-ΔΔCt^ method [56].

### Statistical analysis

Statistical analyses were carried out using R statistical software version 3.2.3 [57]. The differences in development time, pupation rates, adult emergence rates, fecundity, hatchability and gene expression levels between the controls and treated groups were compared using two-sample Student’s t-test. Graphs were designed using GraphPad Prism 7.01 for Windows (GraphPad Software, San Diego, USA). Data are presented as Mean ± SD of experimental replicates. A *p* value of less than 0.05 was considered statistically significant.

## Results

### Sublethal dose determination and effect on larval survival rates

Following exposure of L3/L4 instars to different concentrations of PA-rich fractions, mortality rates were recorded every 24 h for 48 h in both treated and control groups. The control group had 100% survivorship for the entire experimental period. The half-maximal lethal concentration (LC_50_) was determined using the *dose*.*p* function in the MASS package of R based on fitting a non-linear regression model. At 24 h and 48 h post-exposure, the LC_50_ was found to be 5.52 ppm and 4.45 ppm respectively (Fig 1a). After exposing the L3/L4 instars to doses below the LC_50_ of 5.52 ppm, the survival rates were computed relative to control groups. The proportion of survival in larvae exposed to different concentrations of PA-rich fractions was determined using the Kaplan-Meier method [58] and presented in Fig 1b. Mosquito larvae exposed to 1 ppm exhibited the longest survival period followed by 2.5 ppm, negative control (due to pupation and adult emergence) and 5 ppm, in decreasing order (Fig 1b). Pairwise comparisons between the mean survival times revealed significant differences among the treatments (Log Rank test, *p* < 0.001).

### Sublethal doses of proanthocyanidin-rich fractions disrupted *An*. *gambiae* (*s*.*s*.) larval development and delayed adult emergence

**Fig 2:**
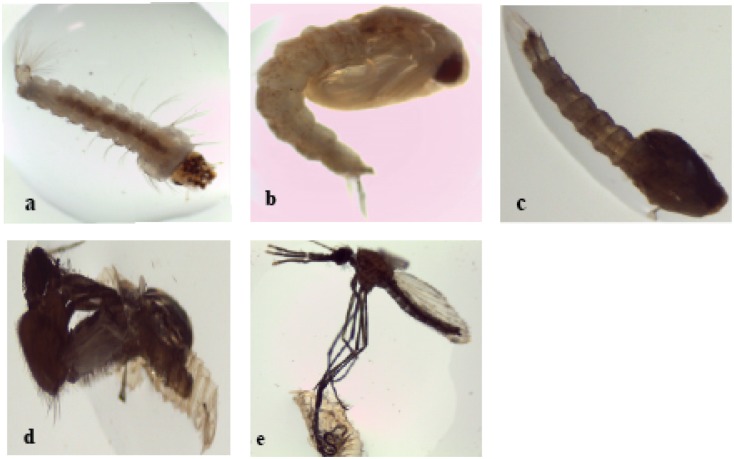
Phenotypic aberrations exerted by proanthocyanidin-rich fractions on *An*. *gambiae* (*s*.*s*.) larval development. Exposure to green tea proanthocyanidin-rich fractions at sublethal doses disrupted larval development inducing abnormal larval-pupal intermediates and prevented adult emergence with mouthparts, wings and legs stuck within the pupal caste. **a**: Normal larvae, **b**: Normal larval-pupal intermediate, **c**: Abnormal larval-pupal intermediate, **d**: Disrupted adult emergence with mouthparts, wings and legs stuck within pupal exuvium, **e:** Failure in adult emergence showing legs stuck in pupal caste. Analysis was performed under a light microscope at magnification of 25×.

In reference to Table 2 below, exposure of *An*. *gambiae* (*s*.*s*.) larvae to PA-rich fractions below the half maximal lethal dose (LC_50_ 5.52 ppm) (Fig 1a) in the range of 1 to 5 ppm exhibited a protracted developmental period in a dose-dependent manner relative to control group. Treatment with 5 ppm significantly extended the mean larval development time to 144 ± 1.92 h (t = -57.39, df = 8, *p* < 0.001) relative to controls with 52.08 ± 1.17 h (Table 2). Additionally, the exposure induced morphological defects whereby the larvae failed to molt into normal pupae resulting in abnormal larval-pupal intermediates (Fig 2c). The adults died during eclosion with their legs and wings stuck in the pupal caste (Fig 2d and 2e). Although no morphological aberrations were induced, significant variations in mean larval development period were also observed with PA-rich fraction concentrations of 2.5 ppm and 1 ppm (100.8 ± 0.11 h) (t = -4.1425, df = 8, *p* = 0.003) (Table 2). Overall, relative to controls, which took 48–72 h to reach adulthood, the L3/L4 transition to pupal stage took an average of 168–288 h following PA-rich fraction exposure while adult emergence occurred after 144–264 h of exposure (Table 2).

**Table 2:** Sublethal dose effects of proanthocyanidin-rich fractions from *Camellia sinensis* leaf extract on development of *An*. *gambiae* (*s*.*s*.) mosquitoes.

Treatment	Mean larval phase time (h)	Pupation rates (%)	Mean emergence time (h)	Successful emergence rates (%)
**Negative control**	52 ± 1.17	47 ± 12.55	72 ± 0.61	63 ± 13.51
**1 ppm**	97 ± 0.33	22 ± 5.70	144 ± 0.45	43 ± 14.83
**2.5 ppm**	104 ± 0.74	11 ± 10.84	192 ± 0.55	8 ± 7.58
**5 ppm**	144 ± 1.92	4 ± 8.94	-	-

Data presented as mean (± SD) of five replicates. Exposure of larvae to *C*. *sinensis* PA-rich fractions significantly extended the larval developmental period, reduced pupation rates and adult emergence (t test, *p <* 0.05). Data on pupation and emergence rates were collected 144 and 192 h after larval incubation respectively. Larval phase and emergence time correspond to h after larval exposure to first pupa transformation and successful adult emergence observation respectively. (-) means no adult emergence observed. Developmental time significantly delayed with increasing PA dosage (t test, *p* < 0.05).

Further, the effect of PA larval exposure on pupation and adult emergence frequencies was investigated and reported in Table 2. Relative to control groups, our findings show significant variations in mean pupation rates when treated with PA-rich fractions at sublethal doses of 1 ppm (t = 4.0555, df = 8, *p* = 0.003), 2.5 ppm (t = 4.8542, df = 8, *p* = 0.001) and 5 ppm (t = 6.2391, df = 8, *p* < 0.001). The variations in mean pupation rates between 2.5 ppm and 1 ppm were not significantly different (t = -2.0083, df = 8, *p* = 0.08). We also established that, given the same duration, the mean adult emergence rates varied significantly between the three treatments (*p* < 0.05). No successful emergence was observed at 5 ppm. A significant reduction in adult emergence was observed when treated with 2.5 ppm (t = 7.9386, df = 8, *p* < 0.001). However, the variations in emergence rates between 1 ppm and control groups were not significantly different (t = 1.9056, df = 8, *p* = 0.09).

### Proanthocyanidin-rich fraction impaired the reproductive viability of female *An*. *gambiae* (*s*.*s*.) mosquitoes

We assessed the reproductive fertility of the mosquitoes that successfully emerged from larvicide-treated water. Equal numbers of emerged adults (males and females) from the respective experimental groups were allowed to mate. The female mosquitoes were blood-fed on mice 72 h post-emergence and the number of oviposited eggs counted under light microscope. Subsequently, the egg hatching viability was assessed by seeding the laid eggs in dechlorinated water and the number of hatched larvae counted every 12 h for 72 h. The counts were compared between the test and control groups and expressed in percentages. The female adults that successfully emerged from larvicide-treated water (1 ppm and 2.5 ppm) exhibited impaired reproductive viability indicated by their inability to lay eggs, unlike in the control group. Egg laying potential of the treated female mosquitoes declined significantly relative to the mosquitoes in the control groups (t = 86.771, df = 8, *p* < 0.001). More than 80% (317/374) of eggs from the control groups hatched normally into viable L1 instars.

### Differential gene expression analysis

**Fig 3:**
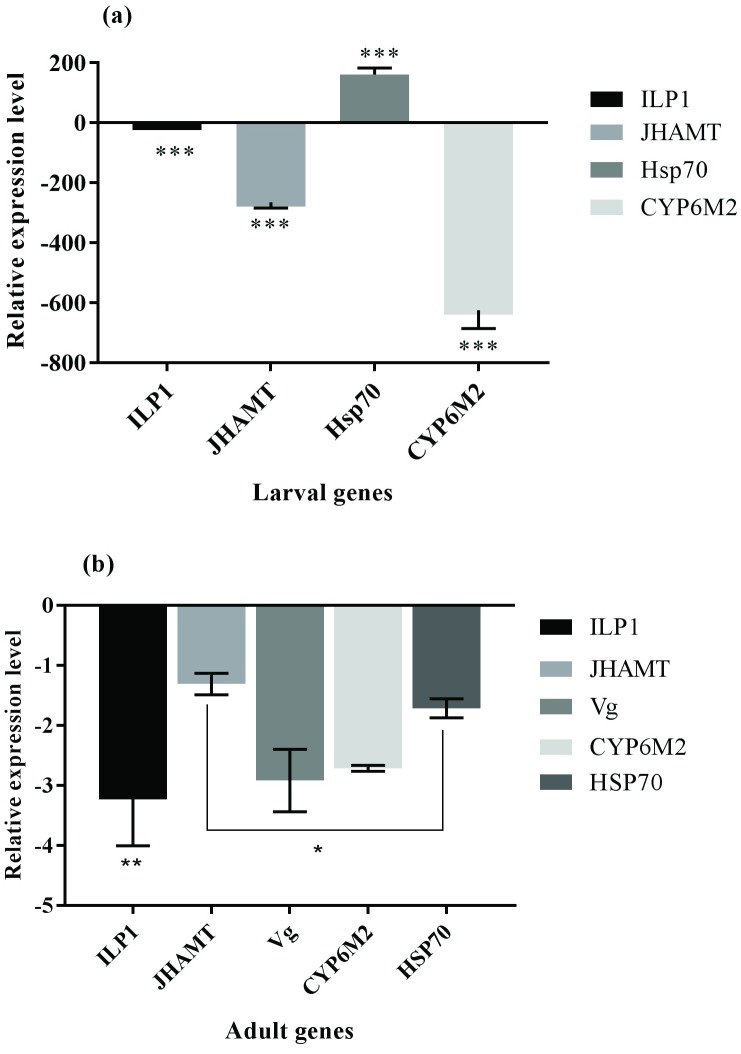
Proanthocyanidin-rich fraction modulated larval and adult genes. Each bar represents Mean (± S.D) fold changes of 3 replicates. **(a):** Treated *An*. *gambiae* (*s*.*s*.) larvae exhibited significant down regulation of genes involved in signal transduction (*AgamILP1*), JH biosynthesis (*AgamJHAMT*) and xenobiotic metabolism (*AgamCYP6M2*) relative to control samples (Student’s t-test, *p* < 0.05). *Hsp70* was observed significantly up-regulated in treated larvae compared to control group (Student’s t-test, *p* < 0.001). **(b):** Emergent female adults from larvicide-treated water demonstrated significant repression of *AgamILP1*, *AgamVg* and *AgamCYP6M2* (*p* < 0.05). *AgamJHAMT* and *Hsp70* were nearly equally down-regulated. Error bars demonstrate variability between replicates. *** Student’s t-test, df = 4, *p* < 0.001, ** *p* < 0.01, * *p* ≤ 0.05.

The phenotypes that resulted from treating developing *An*. *gambiae* (*s*.*s*.) with PA-rich fractions drew our interest to further investigate the molecular basis of the abnormal phenotypes. The genes selected for analysis encode key proteins in JH biosynthesis, signal transduction, xenobiotic metabolism, stress response and vitellogenesis. We established that exposure to sublethal doses of the *C*. *sinensis* PA-rich fraction caused differential gene expression in both larval and adult female genes that appear to be associated with various physiological processes (Fig 3a and 3b). The PA-rich fraction caused significant repression in the expression levels (t = −45.625, *p* < 0.001) of *An*. *gambiae* (*s*.*s*.) larval insulin-like peptide gene 1 (*AgamILP1*), juvenile hormone acid methyltransferase (*AgamJHAMT*) and cytochrome P_450_ family 6 subfamily M peptide 2 (*AgamCYP6M2*) with up-regulation (t = -80.007, df = 4, *p* < 0.001) of 70kDa heat shock protein (*Hsp70*) (Fig 3a). *AgamCYP6M2* was the most down-regulated gene (Δ fold 621.6678 ± 61.09) followed by *AgamJHAMT* (Δ fold 265.0278 ± 18.85) and slightly reduced *AgamILP1* (Δ fold 9.7136 ± 1.42). Transcripts encoding the 70 kDa heat shock protein were significantly up-regulated 159.7863 fold (t = -80.007, df = 4, *p* < 0.001). In female adults, the normalized expression levels of *AgamVg*, *AgamILP1*, *AgamCYP6M2* and *AgamJHAMT* appeared remarkably down-regulated (Fig 3b). *AgamILP1* (Δ fold 3.235 ± 0.77), *AgamVg* (Δ fold 2.92 ± 0.52) and *AgamCYP6M2* (Δ fold 2.715 ± 0.05) were the most affected genes while *AgamJHAMT* (Δ fold 1.31 ± 0.18) and *Hsp70* (Δ fold 1.715 ± 0.16) appeared less repressed (t = -1.4278, *p* = 0.02). Paired t-test between the larval and adult stages showed that the PA-rich fraction significantly perturbed gene expression in larval stages more severely than the adult stages (*p* < 0.001).

## Discussion

Many plant-derived compounds have been investigated for bioactivity against nuisance insects and some reported findings show growth inhibition and sterility-inducing effects [59]. Knowledge of how these effects are mediated at the molecular level remains incomprehensive and limits their potential for full exploitation as ecofriendly bio-pesticides. In the current study, we investigated the effects of sublethal concentrations of *C*. *sinensis* PA-rich fraction, which previously demonstrated larvicidal and growth disruption activities against malaria vectors [54], on *An*. *gambiae* (*s*.*s*.) larval development and reproductive fitness. Our aim was to unravel the possible molecular targets underlying specific phenotypic responses.

Exposure of immature larval stages to sublethal doses of PA-rich fractions exerted profound effects on the developmental timing, adult emergence, female reproduction and egg hatchability. It was therefore speculated that this fraction could have induced impairment of these important hormone-regulated developmental steps and processes. These results agree with the Malaysian report where dengue vectors exposed to sublethal doses of *C*. *sinensis* leaf extracts were found to exhibit an extended larval development time, reduced pupation and adult emergence rates with concomitant reduction in egg output [60]. Under ambient conditions, it takes 11 days on average for *An*. *gambiae* mosquitoes to reach adulthood following egg eclosion through larval stages [61]. However, exposure of *An*. *gambiae* (*s*.*s*.) larvae (L3/L4) to sublethal doses of PA-rich fractions dramatically extended the time taken to reach pupal stages in a dose-dependent manner relative to control groups. In addition, PA-treatment induced abnormal larval-pupal intermediates with reduced survival rates suggesting disturbed developmental events. A similar phenomenon was reported by Lopez *et al*. when *Drosophila melanogaster* larvae were treated with green tea polyphenolic extracts [62]. Sublethal doses of the PA-rich fractions significantly reduced adult emergence rates relative to controls with some failing to eclose from pupal castes. From these findings we inferred that PAs exerted growth reducing effects similar to those exerted by insect growth regulators (IGRs) [63], potentially disrupting metamorphosis in mosquito. Interestingly, the structure of PAs lack similarity to any insect developmental hormone. Thus, PAs could have interfered with hormonal balance *via* other mechanisms culminating into extended larval development period and failed adult emergence.

We further tested the effect of chronic larval exposure to the PA-rich fraction on reproductive fitness of emergent female adults. Results showed that the emerged females from the PA-treated water laid no eggs. This observation could be due to toxicity effects of PAs on female reproductive organs as reported in *D*. *melanogaster* which showed morphological aberrations on exposure to green tea polyphenolic compounds [62]. Observations from Lopez and colleagues showed that the female flies which emerged from (-)-epigallocatechin-3-gallate (EGCG)-treated diet were nearly devoid of mature eggs within the ovaries [62]. Also, the compounds could have interfered with neuroendocrine networks that regulate ovary development, oocyte maturation and egg production in mosquitoes [23]. A plausible explanation for this occurrence is that, polyphenolic compounds modulate endogenous signal transduction pathways, thus interfering with biosynthesis, secretion, transport and metabolism of reproduction-linked hormones [64]. Reduced secretion of regulatory hormones translates to reduced ovarioles and low egg output. Additionally, plant-derived polyphenolic compounds including proanthocyanidins induce embryonic toxicity terminating life prematurely [65].

In order to explain these hormetic responses and gain the molecular insights on the observed phenotypic effects conferred by sub-lethal doses of *C*. *sinensis* PA-rich fractions, differential gene expression profiles were assessed by RT-qPCR. Transcriptional regulation of growth, development and reproduction in insects represent a crosslink between metabolic and hormonal networks [33, 66, 67]. Plant compounds with insect growth regulatory effects have been reported to antagonize the effects of endogenous hormones by dysregulating their biosynthesis, secretion, and binding to their receptors [68, 69]. Exposure of *An*. *gambiae (s*.*s*.) larvae to test compounds dysregulated various genes associated with developmental and reproduction physiology. Gene expression levels of *AgamJHAMT*, an enzyme that catalyzes the rate limiting step of methylation in JH biosynthesis pathway [70], was down regulated, thus correlating with impaired metamorphosis and the extended developmental period of immature stages suggesting that levels of JH were also reduced. In another study, knockdown of JHAMT transcripts in *Ae*. *aegypti* by RNA*i* caused impairment of adult eclosion with concomitant larval mortality [32]. Also, the repression of insulin-like peptide 1 could be responsible for a reduction in JH levels. Down-regulation of *AgamILP1* correlates with repressed expression of *AgamJHAMT* since the two genes co-regulate each other [37]. Depending on the larval nutrient-dependent body size and environmental quality, *ILP1* activates secretion of developmental hormones from their glands [22]. Inputs from these signals may either induce or delay production of developmental hormones depending on the suitability of prevailing conditions [21]. In this regard, ablation of CA has been implicated in slowed development in insects signifying the importance of insulin-like signaling in JH biosynthesis and secretion [71]. Findings from our study suggest that the direct effects of PAs on larval insulin pathways could have impaired JH biosynthesis in the CA. Exposure to xenobiotics induce up-regulation of detoxification enzymes and heat shock proteins [72]. However, CYP6 enzyme encoding genes (*AgamCYP6M2*) were remarkably repressed following larval exposure to the PA-rich fraction. While *CYP6M2* genes are highly expressed in insect midguts and fat body to detoxify xenobiotics [39], the experimental exposure of mosquito larvae to the PA-rich fraction resulted in a dramatic down-regulation of gene expression. Proanthocyanidins astringently precipitate insect midgut proteins eroding their epithelia [49]. Due to their instability under the high pH conditions of insect midguts [49], PAs tend to convert into semi-quinones and highly reactive free radicals which could correspond to the overwhelming reduction in free radical scavenging ability and detoxification potential of CYP6 enzymes. However, the larvae appeared to demonstrate a dramatic tolerance to oxidative stress as suggested by the elevated levels of *Hsp70* that conferred survival. This phenomenon corresponds well with previous reports in *D*. *melanogaster* [62]. Ablation of *D*. *melanogaster* insulin/insulin-like peptide genes through RNA*i* knockdown resulted in remarkable oxidative stress tolerance [73] which supports our findings. Downstream of the IIS pathway is forkhead transcription factor O (dFOXO), a negative regulator of IIS, which controls cellular responses such as stress, cell cycle control, apoptosis, DNA damage and repair [74]. Blockade or repression of IIS translates into unphosphorylated dFOXO, and translocation into the cell’s nucleus induces transcription of genes that enhance longevity and stress tolerance [75].

In any heterogeneous environment, organisms tend to modulate their cellular physiology and genomes to accommodate stress [76, 77]. This is subsequently accompanied by life history trade-offs emergent from resource competition among the various processes [78]. Current knowledge indicates that larval environments are heterogeneous and negatively impact vector physiological fitness [13, 79]. In this regard, transient and/or chronic exposure of developing mosquitoes to insecticides at sublethal dosages has been reported to adversely affect reproduction output and vector performance [80, 81]. In this study, survival from larvicide-treated water selectively decreased the reproductive fitness of emergent female mosquitoes. Reproductive fitness costs have been reported in mosquitoes tolerant to insecticides and toxicants [82]. The expression levels of *AgamVg*, *AgamJHAMT*, *AgamILP1* and *AgamCYP6M2* appeared significantly reduced and were associated with the phenotype. Reduced expression levels of these genes correlated with an absence of eggs from female adults suggesting impaired reproductive fitness induced by the PA-rich fraction. In mosquitoes, egg production is regulated by steroidogenesis in ovaries and vitellogenesis in the fat body through the IIS pathway following bloodmeal digestion [83]. Neuroendocrine secretory cells are then triggered to release ovary ecdysteroidogenic hormone and insulin-like peptides after the bloodmeal that use a cascade of signaling events to induce formation of ovaries and yolk protein biosynthesis by regulating JH [23, 35, 84–86]. Thus, perturbation of this regulated crosstalk *via* either genetic ablation, RNA*i* silencing or insecticide exposure culminates in impaired reproduction [31]. Taken together, these mechanistic observations seem functionally associated with the perturbed physiological processes exerted by *C*. *sinensis* PAs on experimental exposure.

## Conclusion

We assayed the effects of proanthocyanidins isolated from *C*. *sinensis* leaves on *An*. *gambiae* (*s*.*s*.) larval development and female mosquito reproductive fitness. We found that the fraction perturbed larval development and impaired mosquito reproductive fitness through repression of JH biosynthesis, xenobiotic metabolism, and signal transduction responsive genes. These findings could inform either the development of green tea-based biolarvicides or the design of small synthetic ligands that can be used as new larvicides for controlling mosquito populations.
